# The Relevance of Selenium Status in Rheumatoid Arthritis

**DOI:** 10.3390/nu12103007

**Published:** 2020-09-30

**Authors:** Francisco Javier Turrubiates-Hernández, Yolanda Fabiola Márquez-Sandoval, Guillermo González-Estevez, Zyanya Reyes-Castillo, José Francisco Muñoz-Valle

**Affiliations:** 1 Instituto de Investigación en Ciencias Biomédicas, Centro Universitario de Ciencias de la Salud, Universidad de Guadalajara, Guadalajara, Jalisco 44340, Mexico; ln.paco.turrubiates@gmail.com (F.J.T.-H.); guillermo.gonzalezestevez@cucs.udg.mx (G.G.-E.); 2 Doctorado en Ciencias de la Nutrición Traslacional, Centro Universitario de Ciencias de la Salud, Universidad de Guadalajara, Guadalajara, Jalisco 44340, Mexico; yolanda.marquez@academicos.udg.mx; 3 Instituto de Investigaciones en Comportamiento Alimentario y Nutrición, Centro Universitario del Sur, Universidad de Guadalajara, Ciudad Guzmán, Jalisco 49000, Mexico; zyanya.reyes@cusur.udg.mx

**Keywords:** rheumatoid arthritis, selenium, trace elements, nutritional status, inflammation, antioxidants, oxidative stress, selenoproteins, glutathione peroxidase

## Abstract

Rheumatoid arthritis (RA) is an autoimmune and inflammatory disease that can cause joint damage. Among the environmental risk factors, diet plays an important role because it can aggravate or attenuate inflammation. Selenium (Se) is considered an essential trace element since it is a structural component of antioxidant enzymes; however, its concentration can be affected by diet, drugs and genetic polymorphisms. Studies have reported that RA patients have a deficient diet in some food groups that is associated with parameters of disease activity. Furthermore, it has been shown that there is an alteration in serum Se levels in this population. Although some clinical trials have been conducted in the past to analyze the effect of Se supplementation in RA, no significant results were obtained. Contrastingly, experimental studies that have evaluated the effect of novel Se nanoparticles in RA-induced models have shown promising results on the restoration of antioxidant enzyme levels. In particular, glutathione peroxidase (GPx) is an important selenoprotein that could have a modulating effect on inflammation in RA. Considering that RA patients present an inflammatory and oxidative state, the aim of this review is to give an overview of the current knowledge about the relevance of Se status in RA.

## 1. Introduction

Rheumatoid arthritis (RA) is an autoimmune, chronic and inflammatory disease that affects ~1% of the global population [1]. It is characterized by an excessive production of inflammatory mediators including cytokines (IL-1β, IL-6, TNF-α, IL-17), chemokines and autoantibodies that lead to an exacerbated activation of immune system cells. This disease, if not treated properly and in a timely manner, can lead to irreversible joint damage [1,2]. Although the etiology remains unknown, it has been considered as a multifactorial disease resulting from the interaction of genetic, hormonal and environmental factors that contribute to the loss of immune tolerance [3,4,5]. Among the environmental risk factors, diet has been reported to play an important role in the regulation of RA disease activity as specific foods can aggravate or attenuate inflammation [6].

Recently, it has been established that nutrition is essential for the proper development of the immune system, which has led to the study of the relationship between both of them [7]. Thus, immunonutrition has emerged as a new concept that is based on studying the modulation of immune system activity through the intake of specific nutrients in amounts greater than those of a normal diet [8]. Several immunonutrients have been identified, including amino acids, polyunsaturated fatty acids (PUFAs), short chain fatty acids (SCFAs), vitamins and trace elements [7,8]. In particular, the importance of an adequate consumption of trace elements such as selenium (Se), zinc, copper and iron, is supported by their effects on the activation of the immune system for the protection of infections caused by bacteria, viruses and parasites. In addition, these nutrients are cofactors and structural components of important antioxidant enzymes that limit inflammatory activity [9,10].

In this context, although there has recently been an increase in the evidence of the benefits of diet and immunonutrients in RA, a specific nutritional treatment guide for this population has not yet been established, so it remains of special interest to study the effect of dietary patterns and the addition of individual nutrients in the diet of patients with RA, as is the case of trace elements. The relevance of an appropriate consumption of trace elements such as Se is due to its antioxidant and anti-inflammatory effect, since it has been reported that these patients have an increase in the physiological concentrations of reactive oxygen species (ROS) that can cause damage to cellular components, including proteins, lipids and DNA, that promote the destruction of cartilage and bone [11,12,13]. Therefore, the aim of this review is to provide an overview of current knowledge about the relevance of Se status in the pathological course of RA.

## 2. Selenium and Selenoproteins

Se was discovered in 1817 by the Swedish chemist Jons Jacob Berzelius. For years, this element was considered harmful because the excessive consumption of foods rich in Se caused diseases in animals and humans [14]. Nevertheless, it was not until 1957 that evidence of positive biological properties was reported, which resulted in a particular interest in elucidating its benefits. In this way, it was discovered that Se is a component of the active center of enzymes with antioxidant properties [14,15]. Nowadays, Se is classified as a trace element based on its low content in the Earth’s crust (0.05 ppm). Despite the fact that its daily consumption requirement is very low, the World Health Organization (WHO) biological classification groups it into the essential trace elements—this is because its function in living organisms is essential for health [14,16]. This element can be found in both organic and inorganic compounds. Organic compounds include methyl compounds, selenocysteine (Sec), selenomethionine (SeMet) and selenoproteins [14].

To date, 25 selenoproteins have been identified in the human genome, which are the main molecules responsible for performing biological functions; however, only the specific functions of 14 of the identified selenoproteins are well-known (Table 1) [14,17,18,19]. Proteins that contain Sec residues are called selenoproteins. Likewise, selenoproteins that contain a Sec residue in the catalytic position are called selenoenzymes [14]. Sec is considered the 21st naturally occurring amino acid after it was discovered that this amino acid is a major form of Se in the cell and because it is encoded by the UGA codon [18]. Among the best-known functions performed by the glutathione peroxidase (GPx) family is the decrease in hydrogen peroxide (H_2_O_2_) and the reduction in damage by lipid hydroperoxides and phospholipids by converting them to harmless products such as water and alcohols to protect from oxidative, membrane and DNA damage. The expression of some of these selenoproteins such as GPx1 and methionine-R-sulfoxide reductase B1 (MsrB1) decreases with deficiency of dietary Se. Likewise, Se deficiency is reflected in some tissues such as those of the immune system, liver and lungs [18,20,21].

## 3. Biochemistry, Absorption and Metabolism of Selenium

Se is considered a natural semi-metallic trace element found in inorganic (selenite and selenate) and organic form. The organic form occurs when Se competes with the sulfur contained in some amino acids to incorporate them, forming Sec and SeMet. Sec is found mainly in animal proteins, whereas SeMet is found in plant proteins [22,23]. Se, either inorganic (with the exception of selenite) or organic, achieves an absorption above 80% under normal intake and physiological conditions, and occurs primarily in the duodenum and proximal jejunum [23].

**Figure 1:**
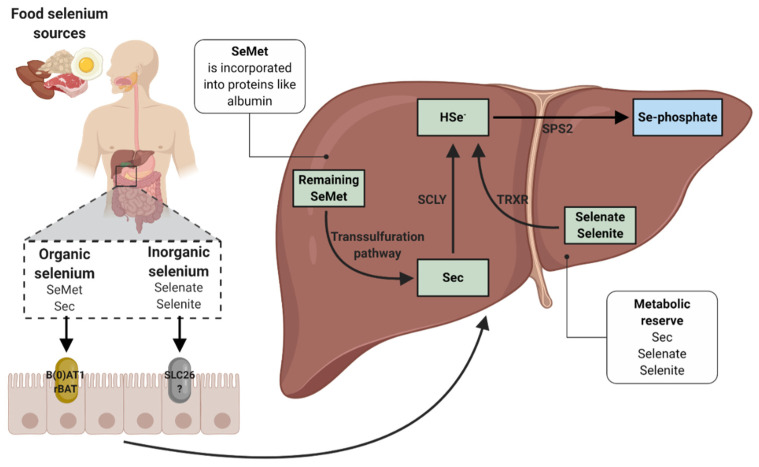
Selenium absorption and metabolism. The organic and inorganic Se sources are absorbed mainly in the small intestine. Organic Se is absorbed mainly through B(0)AT1 and rBAT, whereas inorganic Se is absorbed through SLC26. The liver and other tissues such as muscle and mammary gland are able to capture Se sources from circulation. Sec, selenite, and selenate form a metabolic reserve, whereas SeMet is incorporated into various proteins in place of methionine. The remaining SeMet is converted to Sec via the transsulfuration pathway. The organic Sec sources are converted to HSe^−^ by the SCLY or transselenation, whereas the inorganic Se sources (selenite and selenate) are converted to HSe^−^ by the TRXR in the liver. The glutathione–glutaredoxin pathway is also involved in the production of HSe^−^ from inorganic Se. HSe^−^, through SPS2, is converted to Se-phosphate, necessary for the formation of Sec-tRNA^[Ser]Sec^. The liver synthesizes SeP, which is responsible for the transport of Se through plasma to other tissues (not shown in image). Se, selenium; SeMet, selenomethionine; Sec, selenocysteine; B(0)AT1, sodium-dependent neutral amino acid transporter; rBAT, neutral and basic amino acid transport protein; SLC26, solute carrier 26; SCLY, selenocysteine β-lyase; TRXR, thioredoxin reductase pathway; HSe^−^, selenide; SPS2, selenophosphate synthetase 2; Se-phosphate, selenophosphate; SeP, selenoprotein P.

Although dietary selenium absorption mechanisms remain uncertain, SeMet and Sec have been revealed to be transported by intestinal amino acid transporters, in particular by the sodium-dependent neutral amino acid transporter (B(0)AT1) and by the neutral and basic amino acid transport protein (rBAT), respectively. On the other hand, selenate is absorbed by the solute carrier 26 (SLC26) family of multifunctional anion exchangers [24,25,26]. It has been mentioned that intracellular movement of Se is possibly similar to that of amino acids [26]. Once these selenoamino acids (SeMet and Sec) are absorbed, the liver is able to capture them from the circulation. SeMet is transported to the liver in the form of Se-albumin, whereas Sec and the inorganic forms are transported intact or through a still unknown mechanism (Figure 1) [24]. Hepatocytes can incorporate SeMet in place of methionine in some proteins such as albumin, whereas the remaining SeMet is converted to Sec in the liver and other tissues via the transsulfuration pathway. In this way, the formed and dietary Sec is converted to selenide (HSe^−^) by the action of selenocysteine β-lyase (SCLY). Selenite can be reduced to HSe^−^ through the TRXR in the liver. In addition, the selenite can be converted to HSe^−^ through the glutathione–glutaredoxin pathway. Selenite reacts with glutathione to form selenodiglutathione. Then, selenodiglutathione acts as a substrate for reduction to glutathioselenol by glutathione reductase. Finally, glutathioselenol reacts with glutathione to form HSe^−^ [25]. HSe^−^ will be useful to form the Sec tRNA (Sec-tRNA^[Ser]Sec^) necessary for the production of the selenoproteins required by the body [25,27].

### 3.1. Interactions with Selenium Absorption

One of the main factors influencing the bioavailability of Se in the human body is the chemical form in which it is found. Sec, SeMet and selenate have an absorption efficiency of 70% to 90%, whereas selenite does not exceed 60%. The absorption of this trace element is higher in its organic form through a high protein diet [23,24]. Its absorption is also favored when its intake is accompanied with sources of vitamins A, D and E [28]. In contrast, the absorption of Sec and SeMet is decreased when there is a high consumption on their sulfur analogs (cysteine and methionine), caused by the structural similarity with these amino acids [29,30]. Likewise, other elements such as sulfur, lead, arsenic, and mercury, among others, can be antagonists of Se, so the deficiency of this trace element can cause toxic accumulation of metals [14].

On the other hand, there are also drugs that can interfere with the adequate absorption of Se such as chelators, for example, deferiprone [31]. Although this drug is indicated to remove the excess of iron in thalassemia patients who have received blood transfusions, some studies have reported the alteration in serum Se concentrations that could be due in part to the administration of deferiprone [32,33]. Interestingly, this disease can increase the frequency of the appearance of RA. This may be caused by iron deposition into synovial tissues or to iron chelators such as deferiprone due to the production of free radicals during iron exchange [34].

### 3.2. Incorporation of Dietary Selenium into Selenoproteins

Selenoproteins contain the 21st amino acid (Sec) which is inserted during protein synthesis. Selenoproteins synthesis is initiated when a tRNA^[Ser]Sec^ is charged with serine by seryl-tRNA synthetase (SARS) to form Ser-tRNA^[Ser]Sec^. The seryl residue of Ser-tRNA^[Ser]Sec^ is phosphorylated by phosphoseryl-tRNA^[Ser]Sec^ kinase (PSTK) to be converted to PSer-tRNA^[Ser]Sec^. At this point, the HSe^−^, whether inorganic or organic from the diet, donates a monoselenophosphate which, together with the O-phosphoseril-tRNA^[Ser]Sec^ selenium transferase (SEPSECS), converts the PSer-tRNA^[Ser]Sec^ to Sec-tRNA^[Ser]Sec^ [18,19,35].

**Figure 2:**
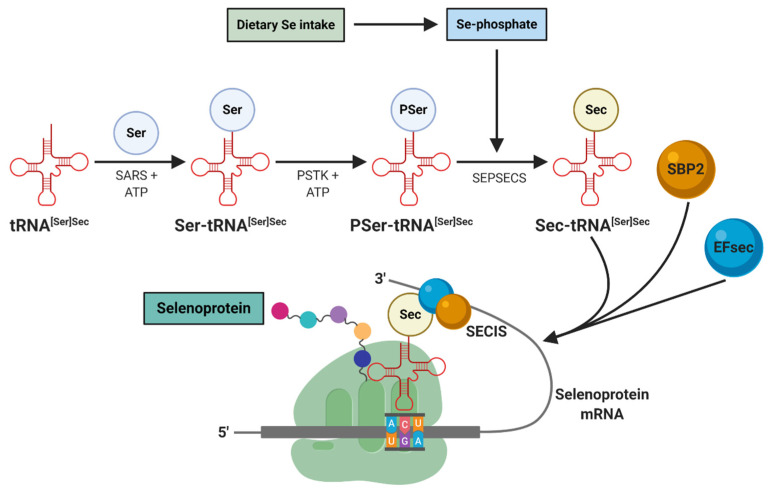
Selenoproteins synthesis. This initiates when a tRNA^[Ser]Sec^ is charged with serine by SARS to form Ser-tRNA^[Ser]Sec^. Afterwards, PSTK phosphorylates Ser-tRNA^[Ser]Sec^ to produce PSer-tRNA^[Ser]Sec^. SEPSECS cooperates with a dietary monoselenophosphate donor to eventually form Sec-tRNA^[Ser]Sec^. The SECIS located in the 3′-UTR region of a selenoprotein mRNA with the support of EFsec and SBP2, form a complex that recruits the Sec-tRNA^[Ser]Sec^, which promotes the recoding of the UGA stop codon. This results in a Sec insertion codon for nascent selenoproteins. Ser, serine; SARS, seryl-tRNA synthetase; PSTK, phosphoseryl-tRNA^[Ser]Sec^ kinase; SEPSECS, O-phosphoseril-tRNA^[Ser]Sec^ selenium transferase; Sec, selenocysteine; Se-phosphate, selenophosphate; SECIS, Sec insertion sequence; 3′-UTR, 3′-untranslated region; SBP2, SECIS binding protein 2; EFsec, specific elongation factor.

This Sec-tRNA^[Ser]Sec^ will be in charge of transferring Sec to selenoproteins. For this, it is necessary that the Sec-tRNA^[Ser]Sec^ decodes the UGA codon as a Sec codon instead of a stop codon. For this mechanism to be carried out, it is essential to have a *cis* element, as is the case of the Sec insertion sequence (SECIS) element in the 3′-untranslated region (UTR) of selenoprotein mRNA that functions as a platform for RNA-binding proteins. SECIS is supported by two *trans*-acting elements: the specific elongation factor (EFsec) and the SECIS binding protein 2 (SBP2). These elements form a complex to recruit Sec-tRNA^[Ser]Sec^, resulting in the recoding of the UGA stop codon to a Sec insert codon [35,36,37,38]. When this occurs, the completion of the protein translation could be through any other stop codon (UAA, UAG or UGA) [18], although some reports indicate that the stop codon is UAA [19,35]. This mechanism promotes nascent protein chains with Sec, which are called selenoproteins (Figure 2) [35].

## 4. Nutritional Selenium Status in Rheumatoid Arthritis

RA is one of the most common autoimmune diseases around the world [39]. This disease is characterized by the altered production of inflammatory mediators that lead to chronic inflammation of the synovial tissue, and eventually if it is not treated in time, cartilage and bone damage occurs [1,2]. Although its etiology remains unknown, the association of genetic, hormonal and environmental factors in RA development has been established [3,4,5]. Among environmental risk factors, evidence has reported that diet is an important modulator of RA activity due to the fact that several nutrients and active compounds in food can interact with the immune response [6]. Thus, although pharmacological treatment of RA patients is currently more effective, not all treatments achieve remission of the disease [2]. Based on these findings, the inclusion of immunonutrients in the diet of RA patients could be an alternative to improve some disease conditions such as pain alleviation, reduced count of tender joints, and shortening of the morning stiffness duration, which can also influence on the attenuation of the disease clinical activity [6].

In this context, recent studies have assessed the nutritional characteristics of this population to identify the nutritional patterns that are associated with disease activity. Observational studies that have evaluated the diet of RA patients using the Healthy Eating Index (HEI), a diet quality index that measures alignment with the Dietary Guidelines for Americans [40,41], have reported lower scores regarding the diet quality compared to healthy control subjects [42,43]. Similarly, other studies that have evaluated the diet quality with the HEI or another dietary assessment tool, reported that a poor diet quality is associated with higher concentrations of C-reactive protein (CRP), a higher erythrocyte sedimentation rate (ESR), and a longer duration of morning stiffness [44,45]. The low scores on the HEI correspond to a low adherence to the recommendations for the consumption of fruits, vegetables, whole grains, fatty acids, seafood, saturated fats, refined grains, added sugars, sodium and empty calories [42,43,44].

Despite studies of this type having been carried out and the tools for evaluating diet having improved, there are few studies that address micronutrient deficiency in RA patients. The importance of the adequate consumption of micronutrients is that they have a fundamental role in the immune system throughout life. Among the micronutrients, vitamins A, C, D, E, B2, B6, B9, B12 and minerals such as iron, zinc and Se are involved in the activation and functioning of the immune response [46]. Se is an essential trace element for human health and exerts its biological functions through selenoproteins. This trace element has antioxidant functions because it is an essential component of GPx, and exerts anti-inflammatory functions by inhibiting the NF-κB cascade and reducing the production of inflammatory mediators [25,47,48].

A study conducted in Iranian women with established RA reported that there was a consumption higher than the Dietary Reference Intakes (DRIs) of Se (87.7 ± 56.2 µg/day); in this study, only 26% of the sample did not reach DRIs of Se [49]. Likewise, another study carried out in Iran also reported higher Se consumption than DRIs, which also showed a negative correlation with prostaglandin E_2_ levels (R = −0.21; *p* =< 0.05) [50]. In contrast to these results, a study conducted in New Zealand reported that RA patients had a consumption of 36 ± 21.9 µg/day of Se. In this analyzed population, only 6% reached 100% of the DRI of Se (55 µg/day) [51]. Similar to this last result, a cross-sectional study carried out in female patients with RA from Brazil reported a Se consumption of only 24.48 µg/day [52]. On the other hand, a study carried out in Sweden reported that RA patients assigned to a Mediterranean diet had better Se consumption compared to a control diet (56 ± 17 µg/day vs. 39 ± 15 µg/day; *p* = 0.004) [53]. A prospective cohort study in Denmark did not mention the amount of Se consumed; however, it was reported that there was no association between the amount of this element in diet and the incidence of RA (IRR = 1.00 [95% CI: 0.98–1.01; *p* = 0.51]) [54]. Another study of this type in the Finnish population from which information on the status of rheumatoid factor (RF) in 122 individuals was obtained, reported that a higher concentration of Se may be a protective factor against the development of RA in individuals seronegative to RF (RR = 0.16 [95% CI: 0.04–0.69]) [55]. Contrary to this result, a study in a sample of 152 women who developed RA (62% RF-seropositive) after a follow-up of almost 11 years in the United States (*n* = 29,368) did not demonstrate a protective association against the risk of RA in users of the consumption of supplemented Se (RR = 0.63 [95% CI: 0.32–1.23; *p* = 0.17]) (11 users vs. 141 non-users) [56].

The results of the previous studies in relation to dietary consumption of Se are not consistent, and this could be explained by several aspects. One of them is that the concentration of Se in food is variable with respect to the region around the world where the food was produced [14], so the food composition reference tables on which the analyses were based could vary between studies. Ideally, each country should have its own food composition table and be freely informed of them in order to estimate and compare Se consumption among the different studies. Likewise, the lack of studies reporting dietary intake of Se could be due to the limited information on the concentration of this trace element in food. Another point to consider is that the food frequency questionnaires and 24 h dietary recalls could underestimate the consumption of nutrients. In fact, in some prospective studies, the questionnaires were applied only at the beginning [54] without considering that diet can be variable over time. Furthermore, the follow-up time may not have been sufficient for the probable development of RA. Nevertheless, these tools for evaluating habitual food consumption are still some of the most appropriate for this type of study. It is also important to say that most of the studies mentioned here did not have a comparison control group [49,50,51,52,54,56]. In contrast, there are also studies in which the consumption of Se can be overestimated, and this could be due to the fact that patients with previous knowledge of the recommended foods could over-report their consumption. It is important to mention that some anti-inflammatory and anti-rheumatic medications are likely to cause dyspepsia, loss of appetite, nausea and alteration of taste, which could alter the diet of these patients [51]. Finally, there are no studies that have evaluated both the dietary intake of Se and its serum concentration. This is of utmost importance because some selenoproteins are dependent on the dietary intake of Se, and their concentration in the human organism could be affected by the interaction in the absorption with medications, as well as by the inflammatory state of the disease.

### 4.1. Clinical Trials with Selenium in the Treatment of Rheumatoid Arthritis

In contrast with the observational studies presented above, clinical trials of supplementation with Se have also been carried out in patients with RA, this in order to elucidate its possible beneficial effects on the disease. These studies were based on the hypothesis that supplementation with Se, being an essential component of selenoproteins such as GPx, TRXR and SeP with antioxidant and anti-inflammatory functions, could help to improve some clinical parameters of RA [57,58,59,60,61]. Nevertheless, there are few clinical trials that have evaluated supplementation with Se in these patients, in addition to the fact that these studies are not recent, so the methodology with which they were performed does not align with current standards for the execution of clinical trials. A systematic review carried out in 2007 (Table 2) reported that among the deficiencies of the included studies, there are: the lack of statistical models for the analysis of data between the experimental groups and the placebo groups, the absence of parity in the severity of the disease of the patients and the lack of information regarding the randomization and blinding techniques used in the studies [62]. Therefore, it could be concluded that the lack of rigorousness of these studies does not completely resolve the hypothesis of the possible beneficial effects of Se on RA.

### 4.2. Preclinical Studies with Selenium Nanoparticles in the Treatment of Rheumatoid Arthritis-Induced Models

Recently, some preclinical studies with Se nanoparticles (SeNPs) have been carried out with the aim of proposing complementary therapies in RA. These studies have been conducted on the basis that the dosage and chemical form of Se is important, since it has been shown that high doses of Se compounds (organic and inorganic) or Se enriched yeast can cause toxicity [63,64,65]. Furthermore, it has previously been shown that ROS production is involved in RA pathogenesis by inducing an oxidative state that activates the inflammatory processes of the joints causing tissue damage in RA patients [66]. For this reason, supplementation with SeNPs could counteract the oxidative stress through the activation of antioxidant enzymes. Oral administration of SeNPs (500 µg/kg/day) for 21 days improved the levels of antioxidant enzymes (GPx, superoxide dismutase and catalase) in different tissues. In some cases, animals treated with SeNPs showed significant restoration of these enzyme levels compared to those treated with prednisolone (10 mg/kg/day) [63]. Three years later, this result was replicated by another research group, where an increase in the expression of antioxidant enzymes was also shown (GPx1 and catalase) [64]. In another study, the design of polypeptide composite SeNPs (0.5 mg/kg^−1^, ‘Se@RuNPs’) showed anti-inflammatory effects. The administration of Se@RuNPs induces the generation of nitric oxide synthase (iNOS) and stimulates endogenous nitric oxide (NO). NO phosphorylates AMPKα causing inhibition of mTOR [65], previously described as an important promoter of synovitis and structural damage in RA [67]. The inhibition of mTOR results in the improvement of autophagy flux and the decrease in the phosphorylation of NF-κB, thus reducing the synthesis of proinflammatory cytokines such as TNF-α, IL-6 and IL-1β. Likewise, it was mentioned that Se@RuNPs could inhibit the growth of new vessels of the proliferating RA synovium since the accumulation of NO induced apoptosis of the human umbilical vein endothelial cells (HUVECs), which are commonly used for the study of angiogenesis [65]. These results suggest the potential effects of SeNPs supplementation on RA therapy.

### 4.3. Polymorphisms Related to the Availability of Selenium and Selenoproteins in Humans

In Hartnup disease, an amino acid transport disorder caused by variations in *SLC6A19*, the gene responsible for encoding the transporter of neutral epithelial amino acids B(0)AT1, single nucleotide polymorphisms (SNPs) have been identified; they decrease the transport activity when expressed alone or co-expressed with the angiotensin converting enzyme 2 (ACE2); however, these SNPs are characteristic of the presence of this disease [68]. On the other hand, the SNP D173N (rs121434346) was reported to reduce the transport activity of B(0)AT1 by ~50% [69]. Likewise, in the *SLC3A1* gene responsible for encoding rBAT, more than 128 mutations have been identified, of which 93 have been described as causing cystinuria where one of the most frequent is the T216M SNP (rs369641941) [70]. In 1994, the SNP M467T (rs121912691) was reported to reduce amino acid transport by up to 80% [71]. Under the premise that these transporters have been identified as potential effectors of the absorption of selenoamino acids, the presence of these SNPs could impact the bioavailability of Se in the human organism. Therefore, the study regarding this context could be an interesting area to explore.

More directly, it has also been described that the expression and activity of some selenoproteins can vary depending on the SNPs. In 2012, a study in the *GPX1* gene reported that the C/T genotype (rs1050450) exhibits better GPx1 activity when there is an adequate serum concentration of Se (110.7 ± 1.2 µg/L) (R = 0.244; *p* = 0.0003). On the other hand, there is a trend in the decrease in DNA damage in carriers of the C/C genotype when they have a serum concentration up to a level of 116.07 µg/L (*p* = 0.044), whereas carriers of the T/T genotype *GPX4* (rs713041) have a serum concentration of up to a level of 149.23 µg/L (*p* = 0.042) [72]. In a study carried out in Brazil (*n* = 116), carriers of the G/C genotype of the SNP (rs8179169) in *GPX1* gene had a lower concentration of erythrocyte Se: 38.1 µg/L vs. 63.8 µg/L of the G/G genotype (*p* =< 0.001). In the linear regression analysis, it was reported that the erythrocyte Se is influenced by the plasma Se (used as a marker of Se intake): R^2^ = 0.25; β = 0.05; *p* =< 0.001 [73]. Three years later, this same research group showed that supplementation with Brazil nuts (the main source of Se in the world) for 8 weeks in carriers of the C/C genotype (rs1050450) increased mRNA expression of the *GPX1* gene (*p* = 0.026), whereas that of the T allele did not change. Likewise, *SELENOP* mRNA expression was higher in carriers of the rare A allele for rs7579 before or after supplementation (*p* =< 0.05) [74]. In addition, there are other studies that have reported similar results in relation to the concentration and activity of the GPx isoforms and SeP influenced by Se supplementation [75,76]. These results suggest an impact of genotypes on the circulating concentrations of this trace element, while, at the same time, these concentrations are closely related to the activity of selenoproteins. In this sense, it would be of great importance to determine the genotypes of patients with RA before an intervention with any source of Se, which would help to assess the existence or not of the possible effectiveness of it.

## 5. Serum Selenium Status in Rheumatoid Arthritis

More than 20 years ago, a review of 36 studies from different countries consisting of 7502 apparently healthy individuals proposed that the values considered normal for serum Se concentration should range between 39.37 and 196.85 µg/L [77], after considering that the concentration of Se in food varies according to its content in the soil and the ability of plants to absorb this element in different regions of the world [78]. Nevertheless, the concentration of Se in plasma or serum required for maximum selenoproteins expression has been reported to be 80 to 90 µg/L in healthy individuals [79]. In this way, the serum Se concentration is considered a good marker to determine the status of Se in the human organism [80].

Previously, decreased serum concentration of trace elements has been described as a common occurrence in autoimmune diseases [81,82,83]. Nevertheless, although it has not been clarified whether Se deficiency is a cause or consequence of the development of these diseases, its deficiency could be a factor for the progression of RA, because it has been associated with the generation of ROS and the associated inflammatory state [84]. Several studies that evaluated the serum concentration of this trace element in patients with RA reported lower concentrations compared to that of apparently healthy individuals (Table 3) [55,85,86,87,88,89,90,91,92,93,94,95,96,97,98,99]. Although in most studies the serum Se concentrations of RA patients are within the recommended ranges, there is no doubt that this phenomenon is a feature of the disease.

Recently, a meta-analysis of these studies [100] reported a significant difference in serum Se between RA patients and healthy controls: SMD = −1.04 (95% CI = −1.58 to −0.50, *Z* = −3.77, *p* =< 0.001), with an *I*^2^ = 95.6%; Q = 343.55, *p* =< 0.001. In the meta-regression analysis, it was determined that the year of publication, the sample size, age, sex, and duration of the disease did not impact the serum Se concentration; however, steroid administration was shown to be positively related to serum Se concentration (β = 0.041, 95% CI = 0.002–0.079, *Z* = 2.08, *p* = 0.037). In a sub-analysis, it was found that the serum concentration of Se of RA patients is different across the continents, so it is suggested that the association between RA and Se is caused by an inadequate consumption of this element; however, it is important that rheumatologists monitor this trace element during patient follow-up due to its biological importance.

### 5.1. Effect of Rheumatoid Arthritis Medication on Selenium Status

Currently, there is greater agreement on treatment protocols for RA patients around the world, mainly based on the administration of non-steroidal anti-inflammatory drugs (NSAIDs), disease-modifying anti-rheumatic drugs (DMARDs), glucocorticoids, and biological drugs [101]. However, 100 years ago, aurotherapy was used in RA patients. This treatment consists of administering gold salts intramuscularly or orally. The purpose of gold therapy is to reduce inflammation through the inhibition of immune cells [102]. Auranofin, an orally administered gold therapy drug, has been shown to block the incorporation of Se into selenoproteins by a yet unknown mechanism that can cause oxidative stress. Although the use of this drug in the treatment of RA has decreased at present due to the multiple advantages of methotrexate over aurotherapy, it is considered important to monitor auranofin doses and Se status in patients under this protocol of treatment [103,104,105]. Another drug in the range of medications for the treatment of RA is D-penicillamine. This drug has been shown in previous studies to be a potent inhibitor of GPx activity. This leads to a possible elevation of free radicals. In this sense, supplementation with Se is recommended for the suppression of the side effects of treatment with D-penicillamine. Nevertheless, like auranofin, its administration has been displaced by methotrexate [106,107]. Regarding prednisolone in the treatment of RA, it was related to plasma depression of Se by an unknown mechanism [108]; however, the lack of information on the duration of administration of prednisolone in these patients precludes the conclusion of this event [109]. In contrast to that described by Peretz et al. [108], a study in 1991 reported that serum concentrations of Se are not affected by prednisone treatment [90], whereas Sahebari et al. [99] reported a positive correlation between serum Se concentrations and doses of prednisolone treatment. Finally, Ma et al. [100], in a meta-regression analysis, showed that the use of corticosteroids was associated with higher levels of Se in RA patients. In regard to DMARDs, patients receiving sulfasalazine show better GPx activity [110], whereas hydroxychloroquine is positively correlated with serum Se concentrations [99]. On the other hand, Önal et al. [96] reported that treatment with sulfasalazine, corticosteroids, NSAIDs, chloroquine or methotrexate did not change serum Se levels, whereas a more recent study showed that monotherapy treatment with methotrexate and the combination of anti-TNF with methotrexate increases serum Se levels in RA patients; however, only methotrexate monotherapy was statistically significant after 6 weeks as well as after 6 months of treatment. The authors of this research concluded that anti-rheumatic drugs appear to increase serum Se concentrations only in individuals with low levels of this element and not in patients who maintain high levels [111]. Although information in this area is limited, some drugs more recently used in the treatment of RA appear to have a resolving effect on serum Se levels in some subjects. Therefore, it is essential to continue studying these interactions to determine the impact of therapeutic combinations in this event. This is necessary, since maintaining or improving adequate concentrations of this trace element has potential positive effects in oxidative stress and inflammation through selenoproteins.

### 5.2. Current Hypotheses that Explain the Decrease in Serum Selenium in Rheumatoid Arthritis

Until today, it has been proposed more consistently that the alteration of the Se concentrations of RA patients is caused by a redistribution of this element from the plasma to the tissues as a defense mechanism, the above, modulated by the exacerbated presence of proinflammatory cytokines [98]. Likewise, it has been suggested that proinflammatory cytokines may induce the production of metal-binding proteins and metallothioneins that sequester this metal ions making its availability for peripheral tissues impossible [94]. On the other hand, inflammation could induce the turnover of selenoproteins that leads to a depletion of Se. Likewise, it has been proposed that the production of acute phase proteins in the liver could inhibit the production of SeP, which is synthesized in this same tissue and is responsible for the transport of Se, causing a decrease in the circulation of this element [111]. Nevertheless, other studies did not rule out the possibility that RA patients are predisposed to malnutrition and therefore to inadequate dietary intake of Se [92,100]. In this context, due to the discrepancy between the authors and due to the variability of this trace element in food and in serum of RA patients between the different regions of the world, as well as the lack of information on confounding variables such as the treatments used in each report, we believe it is important to carry out studies that concomitantly evaluate the serum and dietary concentration of Se in RA patients. The aforementioned studies may allow a more complete determination of the association of Se with the disease activity, given that to date, only the association of serum Se concentration with some disease activity parameters such as CRP and ESR and with the disease activity score has been analyzed, showing inconsistent findings [90,99,111].

### 5.3. Serum Selenium as a Biomarker for Rheumatoid Arthritis

As previously mentioned, the concentration of Se in serum or plasma required for the expression of selenoproteins is 80 to 90 µg/L. In particular, SeP corresponds to the primary transporter protein of Se because it contains up to 60% of this trace element. Therefore, the measurement of serum or plasma Se is considered a reliable biomarker for determining its status [79,80,111]. SeP appears to act as a negative acute phase reactant. The decrease in Se concentration occurs with a slight increase in the CRP concentration (5 to 10 mg/L). Likewise, the systemic inflammatory response has been associated with a reduction of up to 48% in plasma Se concentration when CRP is >80 mg/L [112,113]. It has been suggested that inflammatory cytokines (IL-1β, TNF-α and IFNγ) repress the expression of SeP and that the metabolism of Se could be disturbed during the acute phase reaction [114]. The low concentration of Se can deplete circulating antioxidants and thus exacerbate the inflammatory state of the disease through uncontrolled ROS production [115].

In particular, serum Se levels in RA have been associated with CRP and ESR. Sahebari et al. reported a cut-off value of Se concentration for RA <92.4 µg/L with sensitivity of 72.12% and specificity of 84.62% [99,111]. In addition, it has also been reported that Se deficiency could be a risk factor for the development of cardiovascular disease, which represents the most common cause of premature death in subjects with RA [2,116,117]. In fact, there are reports that indicate that a concentration of Se less than 60 µg/L could increase the risk of cardiovascular mortality [118,119]. In contrast, supplementation with Se (200 µg/day) in combination with coenzyme Q10 reduced cardiovascular mortality [120]. Nevertheless, in Deyab et al., no significant associations were found between serum Se concentrations and cardiovascular risk parameters in patients with RA [111].

In this context, although the evidence seems to indicate a close relationship between inflammation and the alteration of Se levels; it is considered essential to continue with the study of the relationship between its serum or plasma levels and the inflammatory parameters of RA. Monitoring of this variable could be added to the list of biomarkers for assessing the clinical status of RA. Furthermore, it could also be functional in determining the response to treatment, since antirheumatic therapy seems to have an influence in Se homeostasis; however, more studies are needed to elucidate its mechanism.

## 6. Antioxidant and Anti-Inflammatory Effect of Selenium

The role of micronutrients in the functioning of the immune system and on infection was mainly based on vitamin C deficiency and the appearance of scurvy. In 1753, James Lind conducted the first recorded clinical trial where he reported that men with scurvy who consumed citrus recovered more significantly. This discovery emerged as the basis for establishing synergy between micronutrients and the immune system [121]. This has led to the fact that there are currently multiple studies that describe the functioning of different vitamins and minerals on immune cells [46,121]. Se is an essential trace element that, through its incorporation into selenoproteins, regulates both innate and adaptive immune responses [122]. Among the various functions that have been described for this element under physiological conditions are the increase in the production of IFNγ, support in the differentiation and proliferation of T cells, maintenance of antibody levels and production of selenoproteins that act as regulators of ROS during oxidative stress [121].

ROS are oxygen-derived radicals generated in living systems. These include superoxide (O_2_^−^), hydroxyl (OH), peroxyl (ROO), perhydroxyl (HO_2_) and H_2_O_2_, as well as reactive nitrogen species (RNS), such as nitric oxide (NO), nitrogen dioxide (NO_2_) and peroxynitrite (OONO^−^). ROS and RNS are called free radicals and are characterized by the lack of one or more paired electrons in the outermost orbital shell [13]. Under physiological conditions, the production and elimination of free radicals must be kept in balance because they are necessary for cell growth, differentiation, proliferation and apoptosis [13,123]. The elimination of these oxidative species is carried out by antioxidant enzymes such as superoxide dismutase, catalase, glutathione reductase, TRXR and GPx, as well as by non-enzymatic elimination by some vitamins (vitamin A, C, E), β-carotenes and antioxidant minerals (copper, zinc, manganese and Se) [11]. Nevertheless, if their concentrations are increased beyond physiological conditions, they can cause damage to cellular components, such as lipids in membranes, proteins, nucleic acids and DNA. The loss of the balance between the production and elimination of free radicals causes oxidative damage, which is known as oxidative stress. In RA, a five-fold increase in the production of mitochondrial ROS was reported, so it has been shown that oxidative stress is a hallmark of this disease [11,12,13].

**Figure 3:**
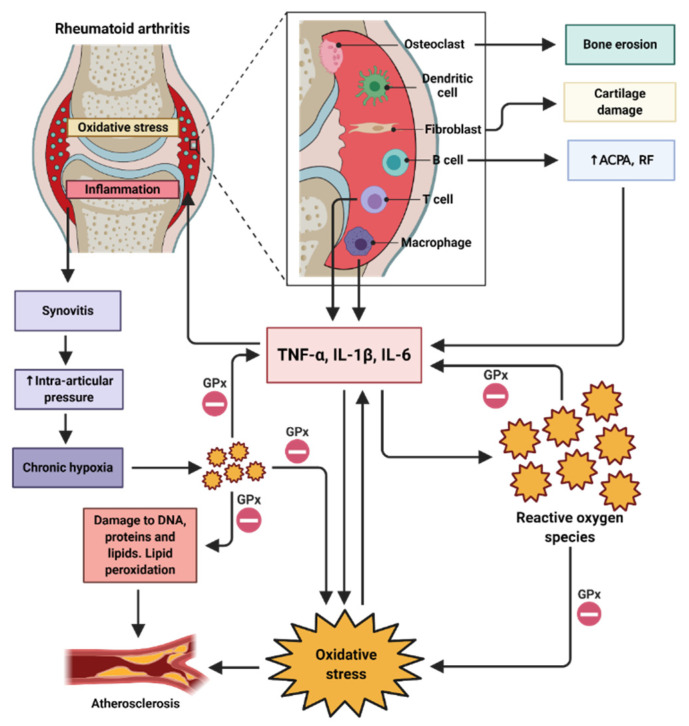
Selenium to promote the resolution of inflammation in rheumatoid arthritis. There is a multidirectional intercommunication between the mechanisms involved in RA. Activation of immune cells in synovial tissue leads to the production of proinflammatory cytokines that have the ability to induce ROS generation. ROS activate NF-κB through phosphorylation of IκB-α or through NIK, generating proinflammatory cytokines that positively feedback the presence of ROS in the joints and lead to the translocation of NF-κB in the other immune cells infiltrated in synovial tissue. Joint pressure generates chronic hypoxia that together with the inflammatory environment causes oxidative stress. Selenoenzyme GPx can reduce inflammation through decreasing one of the most produced ROS (H_2_O_2_) and through the inhibition of IκB-α phosphorylation. The constant lipid peroxidation caused by ROS leads to the formation of atheroma plaques. Atherosclerosis is a feature of chronic inflammation in RA. ACPA, autoantibodies against citrullinated peptides; RF, rheumatoid factor; GPx, glutathione peroxidase.

Oxidative stress is a factor that contributes both to the initiation and maintenance of inflammatory diseases such as RA [12,124]. ROS and RNS have been found in the joints affected by this disease, making these species possible effective biomarkers for monitoring disease progression [125,126]. Previously, the link between the cells of the immune system and the mediators of oxidative stress was established. In particular, macrophages and T cells in their activated form in the synovial membrane produce proinflammatory cytokines such as IL-1β and TNF-α that have the ability to induce ROS production which activate NF-κB [127,128,129], thus causing the increased production of proinflammatory cytokines that positively feedback this same route through canonical pathway and by H_2_O_2_ as an alternative pathway, which causes an oxidative and an inflammatory environment characterized by synovitis and eventual joint destruction [123,130]. Likewise, infiltration of B cells into synovial tissue leads to autoantibody production against citrullinated peptides (ACPAs) and RF that can amplify the production of proinflammatory cytokines through synovial macrophages and subsequently the production of ROS [3,131]. On the other hand, another factor that influences the presence of joint oxidative stress is the constant intra-articular pressure caused by inflammation, which in turn causes chronic hypoxia and thus the generation of ROS in the joints [123,132]. To counteract this oxidative and inflammatory environment, it has been described that, among enzymatic antioxidants, Se plays an important role since it is an essential component for the formation and activity of GPx, which is capable of decreasing ROS levels, especially H_2_O_2_, which represents one of the most produced ROS. Subsequently, the decrease in ROS levels reduces the canonical pathway of NF-κB activation, in which ROS phosphorylate IκB-α for degradation. Likewise, the decrease in oxidative stress limits the non-canonical pathway mediated by the activation of NF-κB inducing kinase (NIK), which is responsible for the phosphorylation of IKKα for the formation of the p52:RelB heterodimer. For this reason, Se is considered beneficial for the resolution of inflammation (Figure 3) [47,123,127,133,134,135].

## 7. Selenium Intake Recommendation and its Rich Sources

The amount of Se both in nature and in the human organism is variable around the world. Previously, regions such as New Zealand, Russia and China reported low levels of this element in the soil and in food that negatively impact their population with Kashin–Beck disease and Keshan disease, whereas Se deficiency is rare in the United States and Canada [28,136]. Maintaining an adequate level of Se in the human body is essential for various functions, including the immune response. The severity of pathologies related to the immune system is influenced by the state of Se, which is dependent on the form it is found in food, as well as genetic polymorphisms, and other factors that interact with its bioavailability as mentioned above. Nevertheless, it is not yet fully understood how supplementation with Se can mitigate diseases related to the immune system [136]. The last update of the DRIs for Se was revised by the Food and Nutrition Board of the United States Institute of Medicine. This update is based on the estimated average requirement necessary to maximize GPx activity. The recommended dietary allowance (RDA) for the adult is 55 µg/day (Table 4), whereas the tolerable upper intake level (UL) is 400 µg/day. Although the majority of the population easily meets these requirements, some countries have higher intake recommendations due to a lower average Se status in their populations (e.g., the U.K. recommends 60 µg/day for adult) [136,137].

As mentioned earlier, Se can be found in food in its different chemical forms, so the best way to achieve RDAs is through diet. Sec and SeMet are the most commonly consumed sources of Se—however, their bioavailability, as well as other factors, depends on the presence of vitamins A, D and E, as well as fat, protein and heavy metals. Among the foods that contain the best amounts of this trace element are nuts, breads, tuna, broccoli, eggs, meat, etc. In relation to the content of trace elements in the water, it has been mentioned that the amount of Se is not significant in most geographical regions (Table 5) [25,48,138].

### Brief Overview of Current Nutritional Approaches in Rheumatoid Arthritis

Although diet has been associated as an environmental risk factor for the progression of RA, it has also been considered as a modifiable risk factor. In this sense, due to the fact that a proper nutritional treatment for RA has not yet been established, currently, there are proposals for a nutritional approach to improve some disease conditions. Weight loss has been reported to play an important role in improving disease activity, since excess adipose tissue leads to the production of inflammatory cytokines and adipocytokines, in addition to the stress exerted on the joints by excess weight [5,139,140]. Another proposal that exists for the nutritional treatment of RA is the Mediterranean diet, which is characterized by a diet rich in olive oil, fish and plant foods. Its beneficial effects on inflammation have been reported to be due to the anti-inflammatory attributes of omega-3 and oleic acid. Nevertheless, its effects on RA remain inconclusive [141,142]. In this same approach to eating patterns, it has been reported that a diet rich in vegetables, fruit and fiber has anti-inflammatory effects; in this context, the impact of the vegetarian diet on RA has been studied. A study reported that the gluten-free vegetarian diet improves clinical variables of the disease, in addition to a reduction in IgG anti-gliadin and IgG anti-β-lactoglobulin antibodies due to decreased exposure of exogenous food antigens [143]. Nevertheless, it is important to mention that the presence of anti-gliadin antibodies is mostly associated with celiac disease [144]. On the other hand, it has been shown that the change in the microbiota can influence inflammation in RA [139,145]. Among other types of nutritional approaches that have been studied in the nutritional treatment of RA, there are the effects of flavonoids, probiotics, antioxidants, and fasting [146,147,148,149]. Similar to the latter, it was recently reported that in *Caenorhabditis elegans*, the Sec mimics the effects of caloric restriction, which has shown benefits in lifespan and in age-related diseases. In this study, Sec was shown to mimic the effects of calorie restriction through SKN-1, the ortholog of nuclear factor erythroid 2-related factor 2 (NRF2) in mammalians, which regulates the expression of antioxidant proteins [150]. Although some of the aforementioned therapies, such as the Mediterranean diet and fasting, have shown favorable effects on some parameters of RA, more robust clinical trials are needed to generalize their recommendation; however, they are still promising proposals for accompanying drug therapy in RA patients.

## 8. Conclusions

Adequate nutrition contributes to the prevention of diseases and is essential for the optimal functioning of the immune system. Previously, it has been established that micronutrients play an important role in the immune response. Based on studies showing that RA patients may present a poor diet quality compared to healthy controls, the relevance of nutritional treatment in the RA approach may be useful for the improvement of some clinical parameters of the disease. Furthermore, it has frequently been evidenced that these patients present a decrease in serum Se levels [100], which is a fundamental component for the formation of antioxidant enzymes. These types of enzymes participate in the regulation of the oxidative and inflammatory state, and it has previously been considered as a factor that affects the disease activity. Nevertheless, it is unclear whether Se deficiency is a cause or consequence of autoimmune diseases [84]. In RA, it has been stipulated with more consistency among the studies addressed in this review, that this phenomenon is caused by inflammation [86,94,96,98,99]; however, other studies suggested that deficiency of this trace element in the diet could be the cause of low levels of Se in serum [55,92,100]. Another aspect to consider is the influence of some factors on the bioavailability and activity of Se, such as the geographical region, medications and genetic polymorphisms. Although some clinical trials have been carried out to analyze the effect of Se in RA patients, the expected results have not been obtained. On the other hand, preclinical studies that have studied the effect of novel SeNPs have shown favorable results. In this sense, it is considered necessary to carry out studies that evaluate both the dietary concentration of Se, as well as its concentration in the serum of this population, so that in a more comprehensive way it can be established whether there is an association between the deficiency of this element and progression of disease activity. Likewise, it is important to carry out more rigorous clinical trials and to carry out experimental studies that help to understand the mechanisms in which Se could improve RA. The monitoring of Se levels in RA patients should be considered. Finally, the indiscriminate use of Se supplements should be taken with caution given its possible toxic effects that occur when exceeding its recommended consumption limits.

## Figures and Tables

**Table 1:** Mammalian selenoproteins and their physiological function.

**Selenoprotein (Abbreviation)**	**Well-Known Function**
Glutathione peroxidases (GPx1, GPx2, GPx3, GPx4, GPx6)	Antioxidant enzymes that reduce H_2_O_2_ and organic peroxides to water and alcohols, respectively
Iodothyronine deiodinase (D1, D2, D3)	Their role is to produce and regulate the level of active thyroid hormone, T3, from thyroxine, T4
Thioredoxin reductases (TRXR1, TRXR2, TRXR3)	They are involved in the regulation of redox reactions in mammalian cells. They are responsible for maintaining the correct intracellular redox potential
Selenoprotein P (SeP)	It is responsible for the transport of Se through plasma to certain tissues
Methionine-R-sulfoxide reductase B1 (MsrB1)	Functions as a methionine sulfoxide reductase
Selenophosphate synthetase 2 (SPS2)	It is required for the synthesis of selenoproteins (including itself) because it produces selenophosphate, the precursor to selenocysteine
**Selenoprotein (Abbreviation)**	**Potential Function**
Selenoprotein H (SELH)	In *D. melanogaster*, it may be involved in antioxidant defense
Selenoprotein I (SELI)	Potential role in phospholipid biosynthesis
Selenoprotein K (SELK)	Involved in calcium flux in immune cells
Selenoprotein M (SELM)	Potential role in protein-folding
Selenoprotein N (SELN)	Possibly involved in early muscle formation
Selenoprotein T (SELT)	Involved in calcium mobilization
Selenoprotein W (SELW)	It may be involved in muscle growth

Adapted from [14,19,21].

**Table 2:** Clinical trials of selenium supplementation in rheumatoid arthritis.

Author, Year [Reference]	Sample Size	Design	Results
Tarp, 1985 [57]	SG: *n* = 20 PG: *n* = 20	6-month follow-up SG: 256 µg/day enriched yeast PG: selenium-deficient yeast	No significant differences between groups
Jäntti, 1991 [58] *Abstract*	*n* = 28	2-month follow-up SG: 150 µg/day PG: not described	‘No clear effect in RA’
Peretz, 1992 [59]	SG: *n* = 8 PG: *n* = 7	3-month follow-up SG: 200 µg/day enriched yeast PG: selenium-free yeast	No between-group comparisons reported
Heinle, 1997 [60]	SG: *n* = 35 PG: *n* = 30	3-month follow-up SG: 200 µg/day sodium selenite PG: not described Concomitant supplementation with fish oil fatty acids (30 mg/kg body) in both groups	No significant differences between groups and not analysis performed in some parameters
Peretz, 2001 [61]	SG: *n* = 28 PG: *n* = 27	3-month follow-up SG: 200 µg/day enriched yeast PG: selenium-free yeast	Significant difference only in two items of a quality of life questionnaire (arm movements and health perception)

Adapted from [62]. SG, selenium group; PG, placebo group; RA, rheumatoid arthritis.

**Table 3:** Serum selenium concentration of rheumatoid arthritis patients and healthy controls.

Author, Year [Reference]	RA Patients		Healthy Controls		*p* Value
	*n*	µg/L	*n*	µg/L	
Aaseth, 1978 [85]	23	93.7 ± 25.2	30	129.13 ± 8.66	-
Hannonen, 1985 [86]	20	75.2 ± 9.2	20	89.6 ± 13.1	-
Borglund, 1988 [87]	7	66.14 ± 8.66	5	77.17 ± 2.36	0.02
Bacon, 1990 [88]	34	99 ± 19	9	109 ± 11	NS
Jacobsson, 1990 [89]	41	76.38 ± 15.75	57	85 ± 13.39	<0.05
O’Dell, 1991 [90]	122	148 ± 42	29	160 ± 25	0.05
Heliovaara, 1994 [91]	14	60 ± 12.8	27	61.2 ± 12.2	0.78
Köse, 1996 [92]	60	107.5 ± 23.76	60	168.45 ± 46.44	<0.001
Knekt, 2000 [55]	122	49.4 ± 12.9	357	50.7 ± 10.2	NS
Witkowska, 2003 [93]	37	64.5 ± 12.18	18	83.9 ± 11	<0.05
Yazar, 2005 [94]	25	64.41 ± 28	25	111.76 ± 67.73	<0.05
Pemberton, 2009 [95]	46	84.55 ± 10.3	58	91.14 ± 12.74	0.003
Önal, 2011 [96]	32	140 ± 37.7	52	166.2 ± 44.3	<0.01
Li, 2014 [97]	60	157.48 ± 49.61	60	192.91 ± 54.33	<0.05
Afridi, 2015 [98]	53	119.45 ± 7.1	52	212.12 ± 8.46	<0.001
Sahebari, 2015 [99]	110	90.92 ± 22.77	100	110.11 ± 18.59	<0.0001
SMD = −1.04 (95% CI = −1.58 to −0.50, *Z* = −3.77, *p* =< 0.001), *I*^2^ = 95.6%					

Adapted from [55,85,86,87,88,89,90,91,92,93,94,95,96,97,98,99,100]. RA, rheumatoid arthritis; NS, not significant.

**Table 4:** Recommended Dietary Allowance for selenium (µg/day).

Age	Male	Female	Pregnancy	Lactation	UL
14–18 years	55	55	60	70	400
19–50 years	55	55	60	70	400
≥51 years	70–100	70–100			400

Adapted from [28,137]. UL, upper intake level.

**Table 5:** Food selenium sources.

Food	Serving	Selenium (µg)	Selenium Compound
Brazil nuts	1 ounce	543.5	SeMet
Fish	3 ounces	92	SeMet/selenite/selenate
Pork	3 ounces	32.5	SeMet/selenate
Chicken	3 ounces	22	SeMet/Sec
Rice	1 cup	19.1	SeMet
Beef	3 ounces	18	SeMet
Whole-wheat bread	2 slices	16.4	SeMet/selenate
Egg	1 large	15	SeMet/Sec
Milk (fat free or skim)	1 cup	7.6	Sec/selenite
Lentils	1 cup	6	SeMet/selenate
Broccoli	1 cup	4.4	SeMet/selenate
Potatoes	1 piece	1.5	SeMet

Adapted from [48,137,138]. SeMet, selenomethionine; Sec, selenocysteine.

## Abbreviations

OH	hydroxyl
ACE2	angiotensin converting enzyme 2
ACPAs	autoantibodies against citrullinated peptides
B(0)AT1	sodium-dependent neutral amino acid transporter
CRP	C-reactive protein
DMARDs	disease-modifying anti-rheumatic drugs
DRIs	Dietary Reference Intakes
EFSec	specific elongation factor
ESR	erythrocyte sedimentation rate
GPx	glutathione peroxidase
H_2_O_2_	hydrogen peroxide
HEI	Healthy Eating Index
HO_2_	perhydroxyl
HSe^−^	selenide
HUVECs	human umbilical vein endothelial cells
iNOS	nitric oxide synthase
MsrB1	methionine-R-sulfoxide reductase B1
NIK	NF-κB inducing kinase
NO	nitric oxide
NO_2_	nitrogen dioxide
NRF2	nuclear factor erythroid 2-related factor 2
NSAIDs	non-steroidal anti-inflammatory drugs
O_2_^−^	superoxide
OONO^−^	peroxynitrite
PSTK	phosphoseryl-tRNA^[Ser]Sec^ kinase
PUFAs	polyunsaturated fatty acids
RA	rheumatoid arthritis
rBAT	neutral and basic amino acid transport protein
RDA	Recommended Dietary Allowance
RF	rheumatoid factor
RNS	reactive nitrogen species
ROO	peroxyl
ROS	reactive oxygen species
SARS	seryl-tRNA synthetase
SBP2	selenocysteine insertion sequence binding protein 2
SCFAs	short chain fatty acids
SCLY	selenocysteine β-lyase
Se	selenium
Sec	selenocysteine
SECIS	selenocysteine insertion sequence
Sec-tRNA^[Ser]Sec^	selenocysteine tRNA
SeMet	selenomethionine
SeNPS	selenium nanoparticles
SeP	selenoprotein P
Se-phosphate	selenophosphate
SEPSECS	O-phosphoseril-tRNA^[Ser]Sec^ selenium transferase
SLC26	solute carrier 26
SNPs	single nucleotide polymorphisms
TRXR	thioredoxin reductase
UL	upper intake level
UTR	untranslated region
WHO	World Health Organization
